# Efficacy and safety of scalp acupuncture for poststroke depression: A meta-analysis and systematic review

**DOI:** 10.1097/MD.0000000000034561

**Published:** 2023-08-04

**Authors:** Wenxi Jiang, Xicheng Jiang, Tianyang Yu, Yang Gao, Yuanzheng Sun

**Affiliations:** a School of Graduate, Heilongjiang University of Chinese Medicine, Harbin, Heilongjiang Province, China; b School of Basic Medical, Heilongjiang University of Chinese Medicine, Harbin, Heilongjiang Province, China; c Department of Acupuncture II, The Second Affiliated Hospital of Heilongjiang University of Chinese Medicine, Harbin, Heilongjiang Province, China.

**Keywords:** meta-analysis, poststroke depression, safety, scalp acupuncture

## Abstract

Poststroke depression (PSD) is a common clinical poststroke complication that adversely affects cognitive and physical function rehabilitation. Scalp acupuncture (SA) can significantly improve somatic dysfunction and emotional disorder in stroke patients. This meta-analysis aims to evaluate the effectiveness and safety of SA in the treatment of PSD.

**Methods:**

We conducted a comprehensive search of multiple electronic databases, including PubMed, Cochrane Library, Embase, Web of Science, China National Knowledge Internet, China Science and Technology Journal Database, Wan Fang Data Knowledge Service Platform, and China Biology Medicine databases until December 20, 2022, to identify randomized controlled trials investigating the efficacy of SA in the treatment of PSD. Two independent researchers screened the literature, extracted data, and assessed the risk of bias in the included studies based on the inclusion and exclusion criteria. We performed a meta-analysis of the eligible literature using RevMan 5.4.1 and Stata 15.0 software.

**Results:**

This study comprised a total of 14 randomized controlled trials, 10 of which used SA and 4 of which used SA in combination with electroacupuncture therapy. The results of the meta-analysis revealed that the effective rate of the SA group was significantly higher than that of the Western medicine group (relative risk = 1.09, 95% confidence interval (CI) [1.02, 1.16], *P* = .008). Moreover, compared to the Western medicine group, the SA group demonstrated significant improvements in Hamilton depression scale scores (mean difference = −2.29, 95% CI [−3.88, −0.70], *P* = .005) and neurological function deficit scores (mean difference = −3.06, 95% CI [−5.91, −0.21], *P* = .04). Additionally, the SA group has a lower incidence of adverse events than the western medicine group (relative risk = 0.12, 95% CI [0.05, 0.29], *P* < .00001).

**Conclusion:**

SA has superior efficacy and safety compared to Western medicine for PSD. These findings suggest that SA could be a promising alternative treatment for the assessed condition. Due to the limited number and quality of the included literature, the above conclusions must be confirmed by additional high-quality research.

## 1. Introduction

Poststroke depression (PSD) is a common complication following a stroke, which is characterized by symptoms such as anhedonia, depressed mood, and reduced interest.^[1]^ The pathogenesis of PSD involves various domains, including neurobiology, anatomy, psychology, sociology, and other domains, although the specific mechanisms are yet to be elucidated. Epidemiological surveys^[2–4]^ reveal that the prevalence of PSD is approximately 31% within 5 years of the onset of stroke, with significant temporal variations peaking 3 to 6 months following the event. Additionally, 1 study found that the incidence of PSD was around 41.1% 3 months after the initial cerebral infarction.^[5]^ The neurobiological mechanisms underlying PSD remain enigmatic, and it is currently posited the etiology of PSD is associated with dysregulation of neurotransmitters, inflammatory reactions, vascular factors, genetic predisposition, and various other potential mechanisms.^[6,7]^ PSD can seriously impact stroke patients rehabilitation and psychological condition, posing a substantial burden on socio-economic and healthcare resources.

Western medical treatments can be categorized into pharmacological and non-pharmacological therapies. Pharmacological drugs mainly involve first-line antidepressants, such as selective serotonin reuptake inhibitors (SSRIs), which have several drawbacks, including numerous adverse reactions, delayed efficacy, and a single treatment mechanism. Non-pharmacological treatments, such as psychotherapy, repetitive transcranial magnetic stimulation, music therapy, and occupational therapy, also have the disadvantages of longer treatment durations, greater costs, and patients frequently discontinue treatment due to intolerance.^[8]^

Acupuncture, as a traditional Chinese medical method, has achieved good clinical efficacy in treating PSD,^[9–12]^ offering the benefits of convenience, cost-effectiveness, no side effects, and significant effectiveness. Acupuncture has the potential to ameliorate depressive symptoms in patients and rats with PSD through the modulation of neurotransmitters,^[13]^ intestinal flora,^[14]^ immune-inflammatory responses,^[15]^ and the hypothalamic-pituitary-adrenal axis.^[16]^ Scalp acupuncture (SA), one of the acupuncture techniques, combines traditional meridian and collateral theory with modern cerebral cortex functional positioning areas and has greater efficacy in the treatment of brain-derived diseases, simultaneously relieving the symptoms of depression and neurological deficits in patients.^[17]^ Studies have substantiated that SA can ameliorate behavioral symptoms, modulate neurotransmitters, and regulate inflammatory factors in rodent models of PSD.^[18,19]^ Therefore, SA is a promising complementary treatment that can treat PSD by modulating various mechanisms. However, SA still exhibits certain limitations, and its effectiveness is predominantly contingent upon the expertise and proficiency of the practitioner. In the design of study protocols, it is common for investigators to mandate that participating physicians possess prior certification in acupuncture expertise to mitigate this limitation.

Although 2 meta-analyses^[20,21]^ have been conducted on the effects of SA for PSD currently, their intervention inclusion criteria were not sufficiently rigorous, their literature searches were not comprehensive, and evaluations of adverse effects and neurological deficits were lacking. Thus, a systematic review of randomized controlled trials (RCTs) comparing SA with Western medicine alone in treating PSD is necessary to summarize the available evidence, evaluate its efficacy and safety, and provide evidence-based support for clinical practice.

## 2. Materials and methods

### 2.1. Study registration and study design

This study was conducted according to the Preferred Reporting Items for Systematic Evaluation and Meta-Analysis statement. It was registered in PROSPERO’s International Prospective Register of Systematic Reviews (Registration number: CRD42022382596).

### 2.2. Inclusion criteria

#### 2.2.1. Study type.

RCTs comparing SA with Western medicine for the treatment of PSD.

#### 2.2.2. Study subject.

Patients with PSD, diagnosed using the International Classification of Diseases, Diagnostic and Statistical Manual of Mental Disorders or Chinese Classification of Mental Disorders criteria, and confirmed by computed tomography or magnetic resonance imaging indicating stroke, without prior history of depression.

#### 2.2.3. Intervention.

The treatment group received SA or SA with electroacupuncture (EA), with no restrictions on point positioning, acupuncture method or needle material. The control group received western medicine. In the same trial, the primary treatment method should be the same for both groups.

#### 2.2.4. Outcome.

The main outcome was effective rates, as measured by the reduction in the Hamilton depression rating scale (HAMD). The additional outcomes included HAMD scores, neurological deficit scores, and adverse events. Outcome indicators were measured at both pretreatment and posttreatment time points.

### 2.3. Exclusion criteria

Non-RCTs: animal experiments, letters, case reports, or review articles; Incomplete data or full text not available; Duplicate studies; Treatment group received measures other than SA or EA.

### 2.4. Search strategy

Two independent researchers conducted a thorough search in several databases, including PubMed, Cochrane Library, Embase, Web of Science, China National Knowledge Internet, China Science and Technology Journal Database, Wan Fang Data Knowledge Service Platform, and China Biology Medicine, in order to gather RCTs on SA for the treatment of PSD. The search period spanned from the databases’ establishment until December 20, 2022. Chinese and English studies were only included due to the availability of academic resources and the language proficiency of the research team. The search was performed using a combination of medical subject headings terms and entry terms. The whole search strategy for PubMed was described at this URL: 382596_STRATEGY_20221209.pdf (york.ac.UK).

### 2.5. Study selection and data extraction

The entire retrieved articles were imported into EndNote X9.3.1 software (https://support.clarivate.com/Endnote/) for management. After the software’s automatic de-duplication, 2 researchers independently performed a preliminary exclusion of articles by reading the title, abstract, and keywords of the articles. After reading the entire text, the articles were reevaluated based on the inclusion and exclusion criteria. All disagreements that arose throughout the process of screening were addressed by a discussion with a third researcher.

Two researchers independently extracted the data from the literature and recorded it in Microsoft Excel 2019. The extracted data were cross-checked, and any discrepancies were resolved through consultation with a third researcher. The extracted data included: the first author, the year of publication, baseline characteristics of the population and interventions, acupoints, outcome indicators, and critical elements of the risk of bias assessment. Researchers could contact the original author by email or phone to obtain missing but crucial study-related information if required.

### 2.6. Quality assessment

Two researchers independently assessed the risk of bias for the included studies and cross-checked the results. The RCT risk of bias assessment tool recommended in Cochrane Handbook 5.1.0 (https://handbook-5-1.cochrane.org/) and the software Revman 5.4.1 was used to evaluate the risk of bias in each study. This evaluation system contained 7 aspects: random sequence generation, allocation concealment, performance bias, detection bias, attrition bias, reporting bias, and other sources of bias. The assessment results were categorized into 3 levels: high risk, low risk, or unclear risk. The third researcher would resolve all disagreements via discussion or consultation.

### 2.7. Statistical analysis

The meta-analysis was conducted using Revman 5.4.1 (https://training.cochrane.org/online-learning/core-software/revman) and Stata 15.0 software (https://www.stata.com/stata15/). The relative risk (RR) and mean difference (MD) served as effect indicators for dichotomous and continuous variables, respectively, with a 95% confidence interval (CI) utilized for both. If *I*^2^ > 50% and *P* < .1, the heterogeneity of the studies is high; otherwise, it is low. If there was no significant heterogeneity among the studies, the fixed-effects model was utilized for meta-analysis; otherwise, the random-effects model was utilized. To investigate the sources of heterogeneity, the subgroup analysis and sensitivity analysis were used. During the sensitivity analyses, data from each individual article were systematically excluded, and the remaining articles data were consolidated to compute the combined effect size, which was subsequently compared to the original results. Subgroup analyses were conducted based on the intervention method (SA vs SA + EA) and needle retention time (>1 hour vs <1 hour). The funnel plots and Egger tests were used to assess publication bias. The level of significance for the meta-analysis was set at *α* = 0.05.

### 2.8. Ethics statement

An ethics statement is not applicable to this study as it is a meta-analysis conducted solely on published literature.

## 3. Results

### 3.1. Literature screening process and results

**Figure 1.**
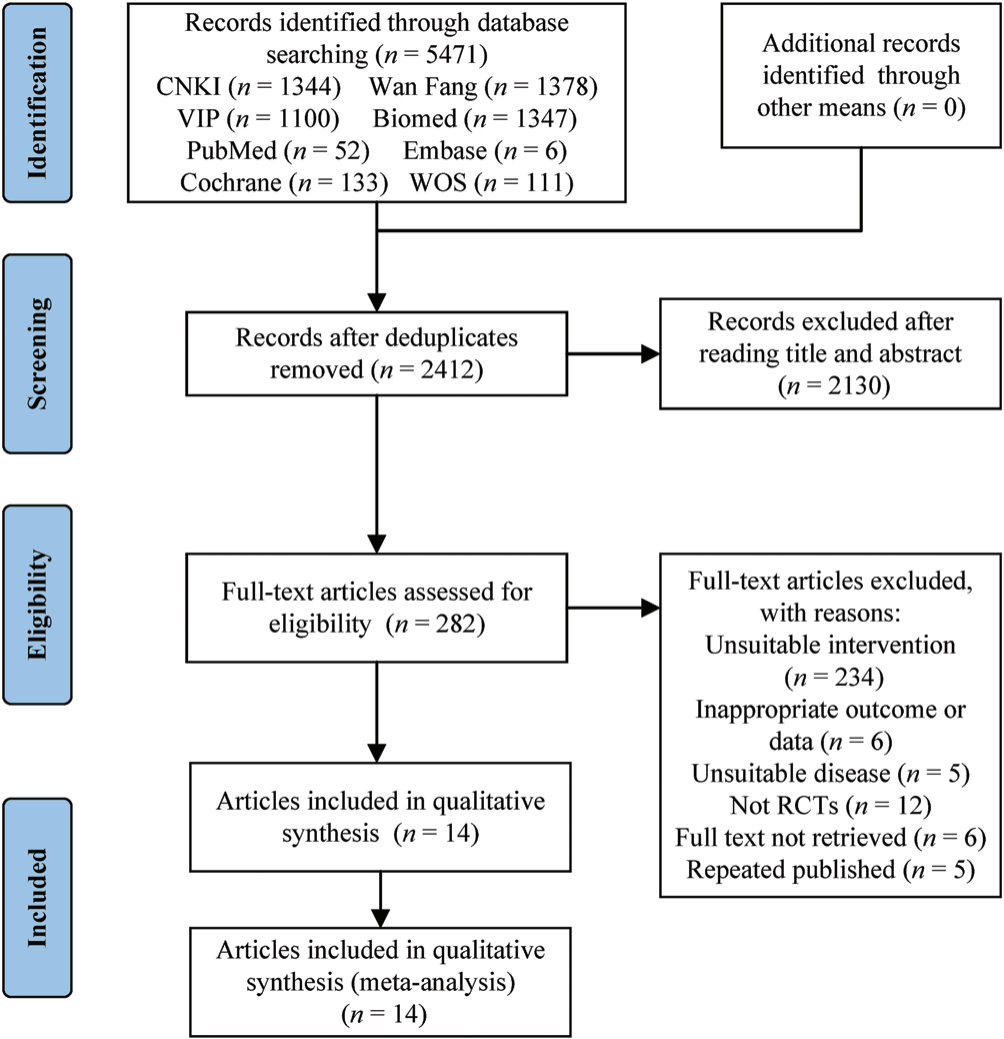
The flowchart of search results for meta-analysis.

Based on the formulated search strategy, 5471 articles were obtained from 8 databases, and 2412 duplicates were eliminated. Following the initial screening of titles and abstracts, 2130 articles were excluded due to their nonconformity with the study criteria. After rescreening the complete texts of 282 documents, we eventually included 14^[22–35]^ papers for meta-analysis, comprising 8 journal articles,^[22,25,29,31–35]^ 1 conference paper,^[26]^ and 5 theses,^[23,24,27,28,30]^ all of which were written in Chinese. The specific literature screening process and results are shown in Figure 1.

### 3.2. Study characteristics

The 1263 patients in the 14 RCTs, all of whom had PSD, were divided into 638 in the treatment group and 625 in the control group. Clinical trials were done in China for all RCTs. The control group was administered western medication, encompassing first-line antidepressants such as Fluoxetine, Sertraline, Venlafaxine, and Clomipramine. The treatment group received SA, with 10 studies^[22,24,25,27–31,33,34]^ using SA and 4 studies^[23,26,32,35]^ using SA in conjunction with EA. Among the outcome indicators evaluated in the studies, 12 studies^[22–24,26–34]^ reported effective rates, 10 studies^[22–25,27–30,33,35]^ reported HAMD scores, 6 studies^[23,25,27,28,30,33]^ reported neurological deficit degree scores and 5 studies^[23,25,27,33,34]^ reported adverse events. A total of 5 studies applied essential treatment, of which 4^[23,25,28,30]^ used conventional therapy in the stroke unit, and 1^[24]^ incorporated psychological rehabilitation. The basic characteristics of the included literature are shown in Table 1.

**Table 1:** Basic characteristics of the included studies.

Author and yr	Sample size (T/C)	Age (yr)	Sex (M/F)	Diagnostic criteria	Intervention	Treatment duration	Needle retention duration	Outcome indicators
Song, et al 1999	29/28	\	\	DSM-III	T: SA C: Fluoxetine	\	2 h	①
Bao. 2016	30/30	T: 35 ± 5.5 C: 36 ± 4.6	T: 8/22 C: 7/23	CCMD	T: SA C: Sertraline	40 d	15 min	①②
Chen. 2009	30/30	T: 62.2 ± 6.2 C: 60.8 ± 6.3	T: 13/17 C: 11/19	CCMD /HAMD	T: SA + EA C: Fluoxetine	4 w	30 min	①②③④
Jia. 2012	30/30	T: 59.9 ± 10.0 C: 56.3 ± 11.3	T: 19/11 C: 18/12	CCMD	T: SA C: Fluoxetine	4 w	6–8 h	①②③④
Sun, et al 2012	30/30	T: 58 ± 7 C: 55 ± 8	T: 19/11 C: 18/12	CCMD /HAMD	T: SA C: Fluoxetine	4 w	6–9h	①②③④
Zhou. 2007	31/30	T: 64.8 ± 5.6 C: 64.1 ± 9.6	T: 20/12 C: 22/12	CCMD	T: SA + EA C: Fluoxetine	4 w	45 min	②
Li. 2021	32/30	T: 54.2 ± 7.6 C: 56.5 ± 6.2	T: 21/11 C: 18/12	CCMD	T: SA C: Sertraline	4 w	30 min	①②③
Jin, et al 2014	32/32	T: 60.6 ± 9.6 C: 61.2 ± 9.3	T: 18/14 C: 20/12	CCMD /DSM-IV	T: SA C: Fluoxetine	4 w	50 min	①②
Jin. 2014	32/32	T: 60.9 ± 9.8 C: 61.6 ± 9.4	T: 19/14 C: 22/13	CCMD	T: SA C: Fluoxetine	6 w	45 min	①②③
Hao, et al 2010	40/36	\	T: 26/14 C: 20/16	CCMD /HAMD	T: SA + EA C: Fluoxetine	30 d	30 min	①
Dong, et al 2003	42/38	\	\	HAMD	T: SA C: Fluoxetine	4 w	30 min	②③④
Dong. 2013	62/61	T: 64.2 ± 10.0 C: 63.2 ± 9.8	T: 32/30 C: 31/30	CCMD /ICD10	T: SA C: Venlafaxine	8 w	30 min	①②
Sun. 2011	80/80	T: 61.5 ± 11.4 C: 62.8 ± 9.6	T: 56/24 C: 50/30	DSM-IV /HAMD	T: SA + EA C: Fluoxetine	4 w	30 min	①
Zhao, et al 2007	138/138	T: 59.2 C: 59.66	T: 76/62 C: 64/74	DSMI-V /HAMD	T: SA C: Clomipramine	4 w	30 min	①④

Outcome indicators: ①Effective rate; ②HAMD; ③Neurological deficit score; ④Adverse event.

C = control group, CCMD = Chinese classification of mental disorders, DSM = diagnostic and statistical manual of mental disorders, EA = electroacupuncture, F = female, HAMD = Hamilton depression rating scale, ICD = international classification of diseases, M = male, SA = scalp acupuncture, T = treatment group.

### 3.3. Risk of bias in included studies

**Figure 2.**
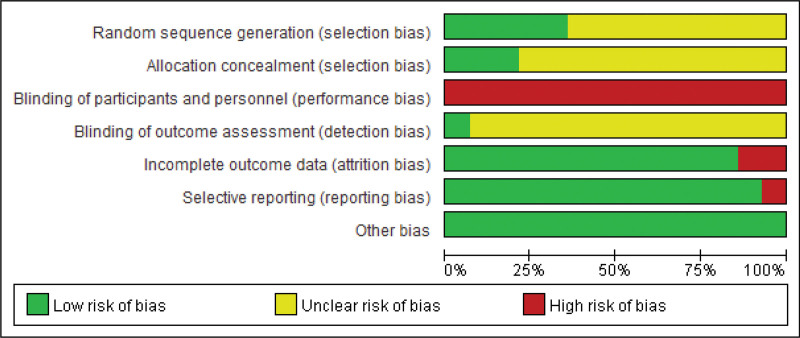
Risk of bias graph.

**Figure 3.**
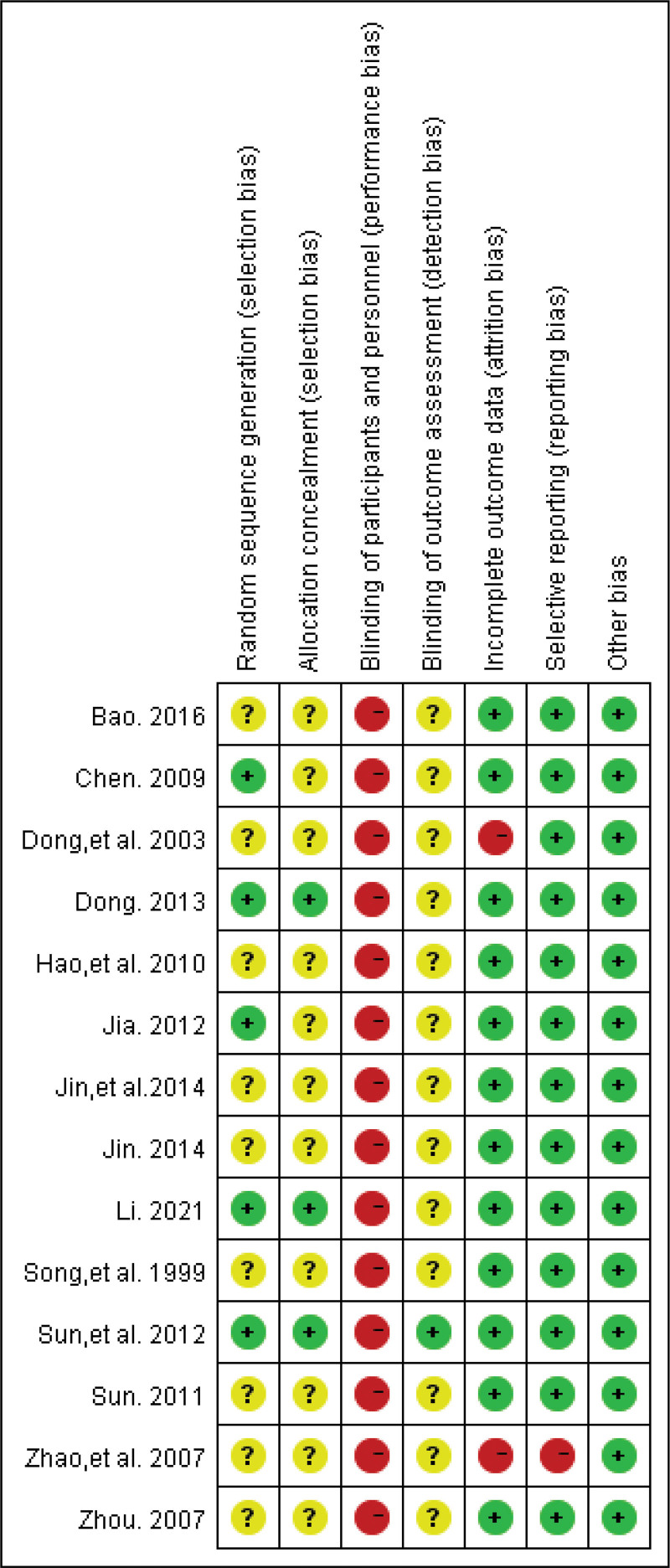
Risk of bias summary.

Based on the evaluation using the Cochrane Collaboration tool, it was revealed that 3 studies^[23,27,30]^ utilized the random number table method, while 1 study^[24]^ employed computerized random number generation. Furthermore, 1 study^[33]^ followed a completely randomized design, whereas the remaining studies failed to specify their randomization strategy. Three studies^[24,30,33]^ implemented allocation concealment, while no mention of allocation concealing techniques was made in the remaining studies. Due to the particularity of acupuncture treatment, it was unfeasible to blind doctors and patients, resulting in all 14 studies being designated as high risk concerning performance bias. Just 1 study^[33]^ attained single-blindness for outcome assessment. Of the 8 studies with no missing outcome data,^[22,23,26,27,29,31–33]^ 4 studies^[24,28,30,35]^ disclosed the missing participants and the reasons for their absence. Furthermore, the number of missing persons in both groups was generally consistent and would not have a significant impact on the results. However, 1 study^[34]^ failed to indicate the number of individuals in the acupuncture group and the control group in Table 1, while another study^[25]^ exhibited significant differences in missing data between the 2 groups, both of which were graded as high risk. The MBI scale, reported in 1 study^[34]^ but not in the protocol, was classified as high risk regarding reporting bias. The risk of bias graph and summary are shown in Figure 2 and Figure 3, respectively.

### 3.4. Meta-analysis results

#### 3.4.1. Effective rate.

**Figure 4.**
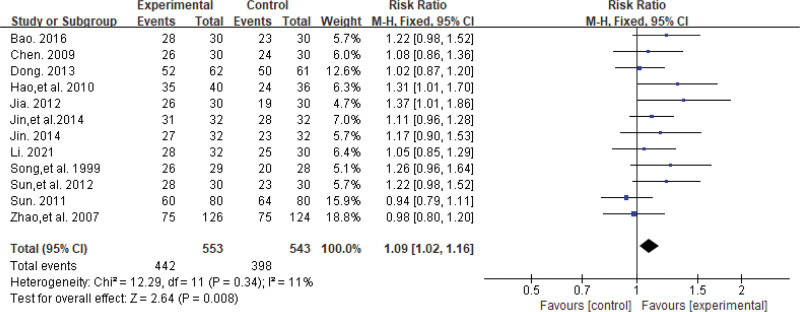
The forest plot of effective rate.

Twelve trials^[22–24,26–34]^ reported effective rates, involving a total of 1096 patients. The results of the heterogeneity test revealed no significant heterogeneity in the data (*P* = .34, *I*^2^ = 11%), and therefore, the fixed-effects model was used to compute the combined effect size for meta-analysis. The results demonstrated a significant difference in effective rates between the treatment and control groups, with SA exhibiting greater effectiveness than Western medicine for treating PSD (RR = 1.09, 95% CI [1.02, 1.16], *P* = .008). The results are shown in Figure 4.

#### 3.4.2. HAMD score.

**Figure 5.**
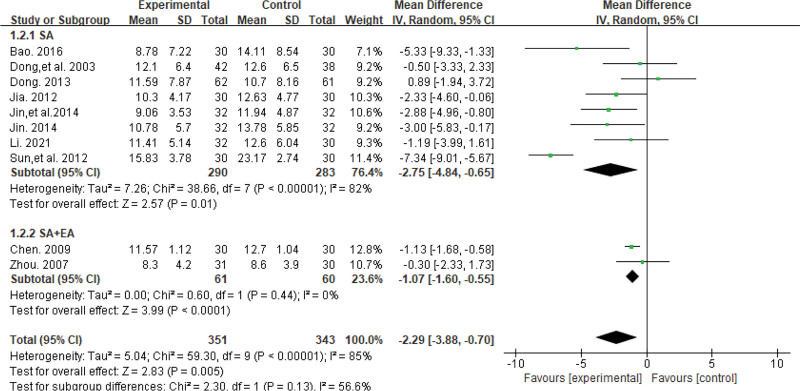
The forest plot of HAMD score. HAMD = Hamilton depression rating scale.

Ten trials^[22–25,27–30,33,35]^ reported HAMD scores involving a total of 694 patients. The results of the heterogeneity test indicated high heterogeneity in the data (*P* < .00001, *I*^2^ = 85%). Thus, subgroup analysis was performed on different intervention methods in the treatment group. Eight studies^[22,24,25,27–30,33]^ were treated with SA, while 2 studies^[23,35]^ were treated with SA plus EA. The results of the heterogeneity test indicated that the SA group still exhibited high heterogeneity (*P* < .00001, *I*^2^ = 82%), while the SA plus EA group demonstrated low heterogeneity (*P* = .44, *I*^2^ = 0%). The combined effect size was calculated using the random-effects model. The findings showed that the change in HAMD score was greater in both the SA and SA plus EA groups than in the western medicine group (MD = −2.75, 95% CI [−4.84, −0.65], *P* = .01; MD = −1.07, 95% CI [−1.60, −0.55], *P* < .0001). The results are shown in Figure 5.

#### 3.4.3. Neurological deficit score.

**Figure 6.**
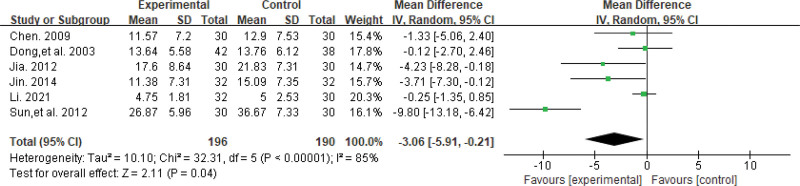
The forest plot of neurological deficit score.

Six studies^[23,25,27,28,30,33]^ reported changes in neurological deficit scores in a total of 386 patients. Among these studies, 5^[23,25,27,28,33]^ used the Modified Edinburgh Scandinavian Neurological Deficit Scoring Scale, and 1^[30]^ used the National Institute of Health Stroke Scale. Due to the high level of heterogeneity observed in the data (*P* < .00001, *I*^2^ = 85%), a meta-analysis was performed using a random-effects model. The results of the analysis showed that the change in neurological deficit score was significantly greater in the SA group compared to the Western medicine group (MD = −3.06, 95% CI [−5.91, −0.21], *P* = .04), and the difference between the 2 groups was statistically significant. The results are shown in Figure 6.

#### 3.4.4. Adverse event.

**Figure 7.**
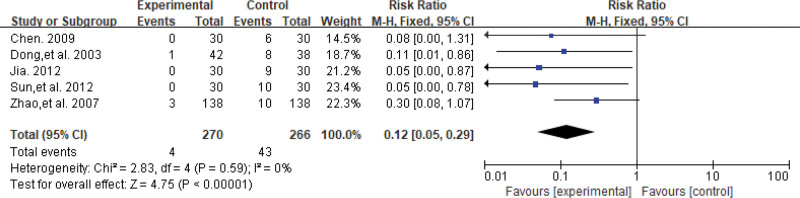
The forest plot of adverse event.

Five studies^[23,25,27,33,34]^ documented adverse events in patients. The results of the heterogeneity test indicated no significant heterogeneity in the data (*P* = .59, *I*^2^ = 0%), hence the fixed-effects model was used for the analysis. The combined effect amount results suggested that SA had fewer side effects compared to Western medicine (RR = 0.12, 95% CI [0.05, 0.29], *P* < .00001), with a statistically significant difference between the 2 groups. The results are shown in Figure 7.

#### 3.4.5. Sensitivity analysis.

**Figure 8.**
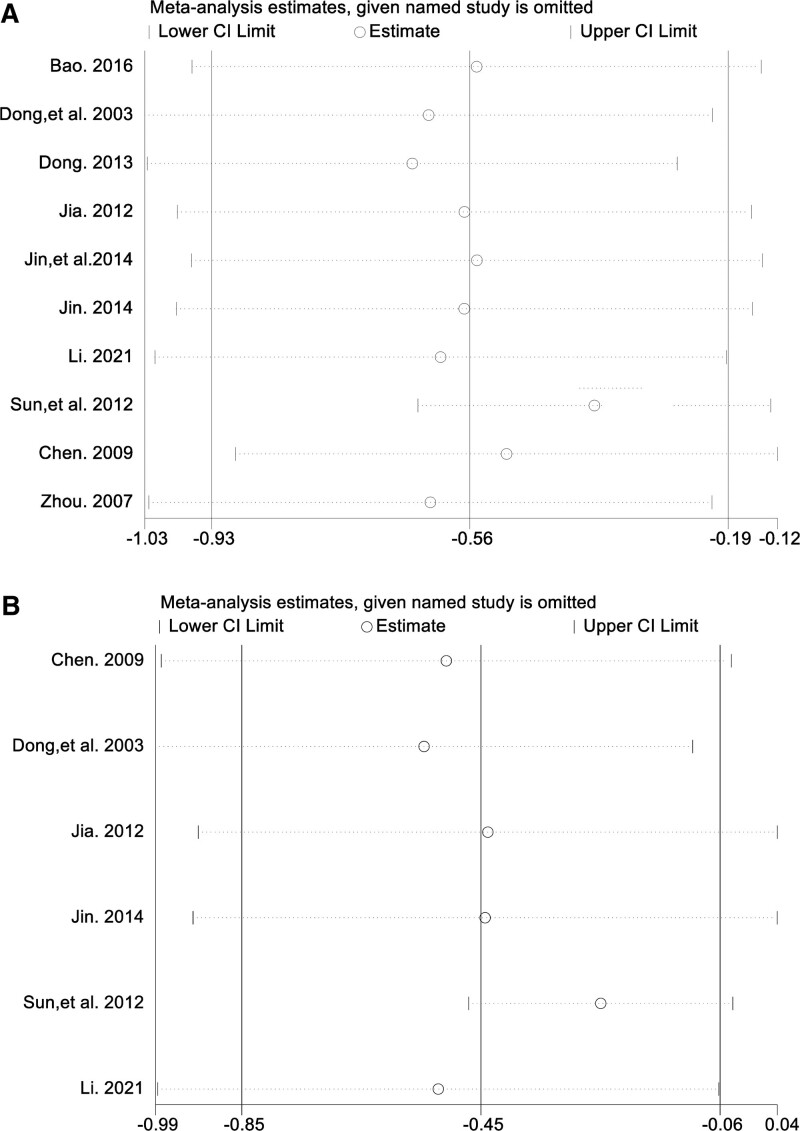
Sensitivity analysis, (A) HAMD score, (B) neurological deficit score. HAMD = Hamilton depression rating scale.

The SA group showed significant heterogeneity in the HAMD scores subgroup analysis (*P* < .00001, *I*^2^ = 82%). Excluding the study by Sun et al^[33]^ greatly reduced this heterogeneity (*P* = .14, *I*^2^ = 34%). The results are presented in Figure 8A. Similarly, there was substantial heterogeneity in the analysis of neurological deficit scores (*P* < .00001, *I*^2^ = 85%). However, excluding the study by Sun et al^[33]^ significantly decreased this heterogeneity (*P* = .16, *I*^2^ = 39%). The results are shown in Figure 8B. In this study, there was a large difference in the scores of the scale scores between the control and treatment groups, potentially leading to a significant deviation of the study from the overall trend observed in the analysis results. This implies that the results of the meta-analysis exhibit lower stability and cannot yield exact conclusions, indicating the presence of potential bias factors.

#### 3.4.6. Publication bias.

**Figure 9.**
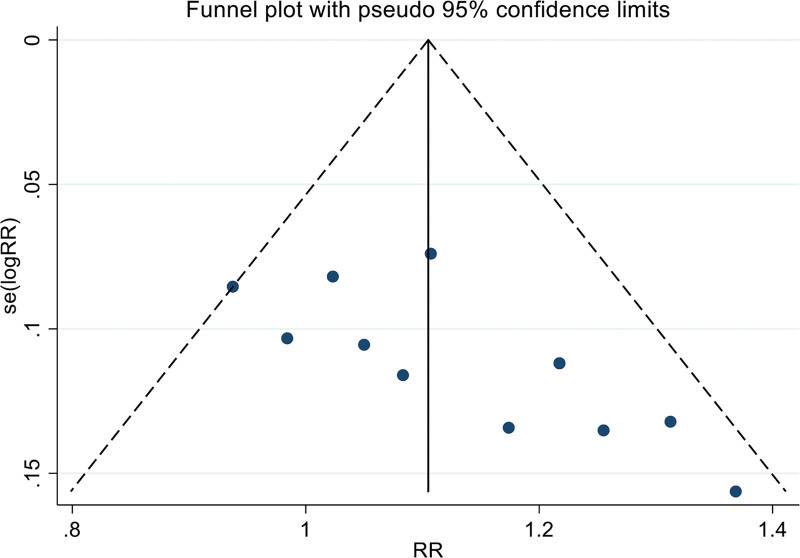
The funnel plot of effective rate.

Based on the effective rates of twelve RCTs, the funnel plot displayed asymmetry, signifying the existence of publication bias. Egger test revealed that twelve of the examined studies were affected by publication bias (*t* = 3.16, *P* = .01). This bias may be due to the fact that all of the included articles were solely written in Chinese, and editors exhibited a preference for studies with superior outcomes. This preference may have further contributed to the incomplete search of gray literature. The result is shown in Figure 9.

## 4. Discussion

In recent years, stroke has emerged as the primary cause of human mortality, with a majority of patients experiencing various complications. Despite this, the current clinical practice focuses more on rehabilitating physical dysfunction rather than addressing psychological disorders.^[36]^ The etiology of PSD remains unclear, with various hypotheses proposed, including the monoamine neurotransmitter, neuroendocrine disorder, nerve signal transduction pathway, intestinal flora dysbiosis, and immune-inflammatory response hypotheses, among others. However, evidence suggests that the incidence of PSD is strongly associated with the severity of neurological impairment, lack of social support, and genetic factors, and is positively correlated with the degree of neurological deficits.^[37,38]^ Moreover, cerebral infarction in the left frontal lobe and left basal ganglia has been identified as a significant risk factor for PSD.^[39]^ SSRIs are recognized as the first-line pharmacological treatment for PSD in China and overseas. The mechanism of action of SSRIs involves inhibiting the reuptake of 5-HT to produce an antidepressant effect.^[40,41]^ However, several prior observational studies have established the efficacy of scalp acupuncture in treating PSD.^[10,11,42]^ Acupuncture has a dual modulatory effect in treating PSD, exerting antidepressant effects through multiple channels, such as the inhibition of neuronal apoptosis, promotion of neurotrophic factor production, regulation of neurotransmitter release, and reduction of inflammatory responses.^[43,44]^ Scalp acupuncture is a vital component of the acupuncture system and is extensively used in clinical practice. Moreover, functional magnetic resonance imaging has revealed that SA can improve motor function and enhance cognitive ability by synergistically modulating several brain regions, such as the cingulate gyrus, cuneus, and precuneus.^[45]^ These findings suggest that traditional Chinese medicine techniques, particularly scalp acupuncture, may hold promise as a potential treatment for depression in poststroke patients, and further research in this area is warranted. Previously published meta-analyses suggest that SA has certain advantages in treating PSD.^[20,21]^ However, based on the limitations of these 2 investigations, this research has presented a meta-analysis of the literature on SA compared to Western medicine in treating PSD and supplemented the latest published RCTs with more comprehensive observation indicators.

In the present study, 2 interventions, namely SA therapy and SA coupled with EA, were applied in the treatment group. The latter intervention involved the application of low-frequency electronic pulse therapy to the head acupoints after needle insertion. The control group, on the other hand, was treated with first-line antidepressants, including Fluoxetine, Sertraline, Venlafaxine and Clomipramine. To assess depressive symptoms and the extent of neurological function recovery, we utilized HAMD and neurological deficit scores. The findings revealed that the SA group exhibited superior clinical effective rates, HAMD and neurological deficit scores, as compared to the Western medicine group. This suggests that treating PSD patients with SA can better alleviate depressive symptoms and promote neurological function recovery, with certain efficacy advantages. SA entails the targeted activation of precise acupuncture points, which exert regulatory effects on the central nervous system and neuroendocrine functions. This form of stimulation possesses the capacity to modulate the levels of neurotransmitters, such as serotonin and norepinephrine,^[19]^ which are pivotal in the regulation of mood. SA has the potential to augment neuroplasticity through the facilitation of neuronal regeneration, synaptogenesis, and angiogenesis.^[46]^ The aforementioned physiological processes facilitate the restoration of neural damage induced by stroke and promote the recovery of signaling pathways associated with emotions. Cultural background and patient preferences may also be among the reasons for the observed superiority of SA in treating PSD. Deriving from the technique of Traditional Chinese Medicine, SA enjoys greater acceptance among Chinese patients, potentially generating elevated patient expectations and contributing to a placebo effect. Furthermore, as a non-pharmacological treatment option, SA caters to patients preferences for natural therapies and avoidance of medication side effects.

EA can partially replace needle manipulation by combining the stimulation of acupuncture with electric current to enhance the curative effect, while making the amount of stimulation controllable and quantifiable.^[47]^ Therefore, in the subgroup analysis of HAMD, we used the intervention of the treatment group as the grouping basis to explore the differences between the SA group and the SA plus EA group. The findings demonstrated that the HAMD score changes in the 2 groups were superior to those in the Western medicine group. However, there was no significant difference between the different subgroups, which may be a consequence of the limited quantity and poor quality of literature in each subgroup. Regarding safety, the incidence of adverse events in the SA group was lower compared to the Western medicine group. Adverse events reported in the Western medicine group predominantly comprised abnormal liver function, dry mouth, palpitations, dizziness, headache, gastrointestinal reactions, and insomnia. Two studies^[25,34]^ documented adverse events in the SA group, wherein Dong et al^[25]^ reported that only 1 patient experienced facial pain, which resolved after symptomatic treatment and subsequent completion of the treatment. These findings suggest that SA has higher safety in treating PSD.

This study had several limitations, and we suggested some recommendations for future research: The limited number of included studies, small sample sizes, and lack of long-term follow-up might have affected the accuracy of the findings. In future clinical studies, there is a pressing need for an increased number of high-quality RCTs with large sample sizes, multi-center collaboration, and long-term follow-up in treating PSD; We searched only Chinese and English literature, and the included studies were all published in Chinese, which may have introduced language bias. This means that the results of the systematic review may not be fully representative of the global system of evidence on the topic. In future endeavors, language constraints in our literature searches shall be eliminated to ensure a more comprehensive and representative analysis of the available evidence; Most studies failed to delineate precise randomization methods and concealment strategies for allocation, and none of them implemented blinding protocols for patients and physicians. This engendered a potential susceptibility to bias in the study outcomes, thereby diminishing the dependability of conclusions. In subsequent endeavors, researchers could be required to refine the specificity of research methods descriptions and enhance peers reviews. To mitigate performance bias in RCTs of acupuncture, it is plausible to establish a sham acupuncture intervention as a control group; The included literature differed in acupuncture depth, frequency of acupuncture, method of needle manipulation, acupoints, and types of Western medicine, which may have contributed to heterogeneity. In the future, RCTs investigating the efficacy of acupuncture for PSD should adhere to internationally recognized uniform standards for trial reporting, such as the CONSORT statement and STRICTA evaluation, to provide high-quality evidence for evidence-based medicine.

## 5. Conclusion

The aforementioned evidence implies that scalp acupuncture may confer more substantial improvements to depression and neurological function in PSD patients, with superior safety and definite clinical efficacy, compared to western medical treatments. Given the constraints of this study, further high-quality RCTs are warranted to verify the clinical utility of scalp acupuncture in the coming years.

## Author contributions

**Conceptualization:** Wenxi Jiang, Xicheng Jiang.

**Data curation:** Wenxi Jiang, Xicheng Jiang, Yang Gao.

**Formal analysis:** Wenxi Jiang, Xicheng Jiang, Tianyang Yu.

**Funding acquisition:** Yuanzheng Sun.

**Investigation:** Xicheng Jiang, Tianyang Yu, Yuanzheng Sun.

**Software:** Wenxi Jiang, Yang Gao.

**Supervision:** Yuanzheng Sun.

**Writing – original draft:** Wenxi Jiang.

**Writing – review & editing:** Xicheng Jiang.

## References

[1] Towfighi A Ovbiagele B El Husseini N. Poststroke depression: a scientific statement for healthcare professionals from the American heart association/American stroke association. Stroke. 2017;48:e30–43. 27932603 10.1161/STR.0000000000000113

[2] Li LJ Yao XM Guan BY. Persistent depression is a predictor of quality of life in stroke survivors: results from a 5-year follow-up study of a Chinese cohort. Chin Med J (Engl). 2019;132:2206–12. 31436596 10.1097/CM9.0000000000000400 PMC6797138

[3] Villa RF Ferrari F Moretti A. Post-stroke depression: mechanisms and pharmacological treatment. Pharmacol Ther. 2018;184:131–44. 29128343 10.1016/j.pharmthera.2017.11.005

[4] Zhang X Zhang Y Liu Y. Effectiveness of mirror therapy on upper limb function, activities of daily living, and depression in post-stroke depression patients. Turk J Phys Med Rehabil. 2021;67:365–9. 34870125 10.5606/tftrd.2021.6635 PMC8606990

[5] Niu Y Sheng S Chen Y. The efficacy of group acceptance and commitment therapy for preventing post-stroke depression: a randomized controlled trial. J Stroke Cerebrovasc Dis. 2022;31:106225. 34837758 10.1016/j.jstrokecerebrovasdis.2021.106225

[6] Blier P. Neurobiology of depression and mechanism of action of depression treatments. J Clin Psychiatry. 2016;77:e319. 27046319 10.4088/JCP.13097tx3c

[7] Wang Z Shi YM Liu FF. Diversiform etiologies for post-stroke depression. Front Psychiatry. 2019;9:761. 30728786 10.3389/fpsyt.2018.00761 PMC6351464

[8] Russo NW Petrucci G Rocca B. Aspirin, stroke and drug-drug interactions. Vascul Pharmacol. 2016;87:14–22. 27765537 10.1016/j.vph.2016.10.006

[9] Chen A Gao Y Wang G. Effect of early acupuncture intervention on post-stroke depression: a randomized controlled trial. Zhongguo Zhen Jiu. 2018;38:1141–4. 30672192 10.13703/j.0255-2930.2018.11.001

[10] Li M Zhang B Meng Z. Effect of Tiaoshen Kaiqiao acupuncture in the treatment of ischemic post-stroke depression: a randomized controlled trial. J Tradit Chin Med. 2017;37:171–8. 29960288 10.1016/s0254-6272(17)30041-9

[11] Ni S Jiang X Peng Y. Tiaoshen Jieyu acupuncture combined with sertraline hydrochloride tablet for post-stoke depression: a randomized controlled trial. Chin Acupunct Moxibustion. 2023;43:19–22. 10.13703/j.0255-2930.20220520-0006 36633234

[12] Yin Z Ge S Huang L. Acupuncture combined with repetitive transcranial magnetic stimulation for post-stroke depression: a randomized controlled trial. Chin Acupunct Moxibustion. 2022;42:1216–20. 10.13703/j.0255-2930.20211221-0002 36397217

[13] Xiao W Zhang XB Wang Z. Effect of manual acupuncture intervention on levels of 5-HTT, 5-HT (1A)R, NEα(2)R of brain tissues in rats with post-stroke Depression. Zhen Ci Yan Jiu. 2016;41:528–34. 29071896

[14] Jiang H Deng S Zhang J. Acupuncture treatment for post-stroke depression: intestinal microbiota and its role. Front Neurosci. 2023;17:1146946. 10.3389/fnins.2023.1146946 PMC10070763 37025378

[15] Wijeratne T Sales C Wijeratne C. A narrative review on the non-pharmacologic interventions in post-stroke depression. Psychol Res Behav Manag. 2022;15:1689–706. 35832139 10.2147/PRBM.S310207 PMC9273151

[16] Le JJ Yi T Qi L. Electroacupuncture regulate hypothalamic-pituitary-adrenal axis and enhance hippocampal serotonin system in a rat model of depression. Neurosci Lett. 2016;615:66–71. 26773866 10.1016/j.neulet.2016.01.004

[17] Xu C Fan G Zhao Y. Comparison and development of different scalp needling schools. Chin Acupunct Moxibustion. 2016;36:663–7. 10.13703/j.0255-2930.2016.06.031 29231468

[18] Cai W Ma W Wang GT. Effect of electroacupuncture on the expressions of serum interleukin-1β, interleukin-6 and tumor necrosis factor-α in the rats with post-stroke depression. Chinese J Integr Med Cardio-Cerebrov Dis. 2019;17:3515–8.

[19] Yangiang Q Hao D Yingchun S. Effects of different acupuncture schemes on neurotransmitters and related inflammatory factors in rats with post-stroke depression. Chin J Rehabilitation Theory Pract. 2023;29:30–7.

[20] Yulong W Yanjiao L Junming A. Meta analysis of head acupuncture therapy for post-stroke depression in the past 10 years. World Chinese Medicine. 2021;16(20):3040–3046.

[21] Huajun Z Linling X Tianshu X. Scalp acupuncture therapy for post-stroke depression:a systematic review. J Liaoning Univ Tradit Chin Med. 2017;19:163–6.

[22] Bao W. Analysis of the effect of treating post-stroke depression with head acupuncture therapy. Contemp Med Symp. 2016;14:29–30.

[23] Chen M. Clinical Observation on Scalp Electroacupuncture for Treatment of Poststroke Depression. Hunan University of Traditional Chinese Medicine. 2009;5:2–14.

[24] Dong B. A randomized Controlled Trial of the Fang’s Scalp Acupuncture Therapy for Poststroke Depression. Shanxi University of Chinese Medicine. 2013;5:8–17.

[25] Dong Z Dai Q Wang F. Comparison of the efficacy of acupuncture and medication for post-stroke depression. Chin J Clin Rehabilitation. 2003;7:3516.

[26] Hao G Ge S. Clinical observation on the treatment of post-stroke depression by cephalic acupuncture of brain toning and penetrating points. Proceedings of the Tenth Academic Conference of the Chinese Association of Integrative Medicine and Western Medicine Psychiatric Committee. Xiamen. 2010;11:72–6.

[27] Jia S. Clinical Observation on Yushi Scalp Clustery Acupuncture for Treatment of Post Stroke Depression. Heilongjiang University of Chinese Medicine. 2012;5:20–5.

[28] Jin S. Clinical Observation on Cranial Sutures Acupuncture for Post-Stroke Depression. Hubei University of Chinese Medicine. 2014;3:22–31.

[29] Jin S Peng L Cheng J. Clinical observation on depression after apoplexy treated by cranial sutures needling method. J Pract Tradit Chin Med. 2014;30:312–3.

[30] Li C. Clinical Observation of Transcranial Repetitive Acupuncture in the Treatment of Post-stroke Depression. Heilongjiang University of Chinese Medicine. 2021;5:14–25.

[31] Song Y Liang H. The efficacy of scalp acupuncture in the treatment of post-stroke depression. Shanghai J Acupunct Moxibustion. 1999;18:8–9.

[32] Sun X. The efficacy of head acupuncture combined with electroacupuncture in the treatment of post-stroke depression. Jilin J Tradit Chin Med. 2011;31:1092–3.

[33] Sun Y Jia S. Clinical study on YU’s cluster needling at scalp acupoints for post-stroke depression. Shanghai J Acupunct Moxibustion. 2012;31:564–5.

[34] Zhao H Zhao W. Clinical study on post stroke depression by acupuncturing baihui point (DU20). Chin Arch Tradit Chin Med. 2007;25:275–7.

[35] Zhou Z. A controlled study of electroacupuncture versus pharmacological treatment of post-stroke depression. China Medical Herald. 2007;16:23 + 128.

[36] Sarkar A Sarmah D Datta A. Post-stroke depression: chaos to exposition. Brain Res Bull. 2021;168:74–88. 33359639 10.1016/j.brainresbull.2020.12.012

[37] Carota A Berney A Aybek S. A prospective study of predictors of poststroke depression. Neurology. 2005;64:428–33. 15699370 10.1212/01.WNL.0000150935.05940.2D

[38] Göthe F Enache D Wahlund LO. Cerebrovascular diseases and depression: epidemiology, mechanisms and treatment. Panminerva Med. 2012;54:161–70. 22801433

[39] Robinson RG Jorge RE. Post-stroke depression: a review. Am J Psychiatry. 2016;173:221–31. 26684921 10.1176/appi.ajp.2015.15030363

[40] Zahrai A Vahid-Ansari F Daigle M. Fluoxetine-induced recovery of serotonin and norepinephrine projections in a mouse model of post-stroke depression. Transl Psychiatry. 2020;10:334. 32999279 10.1038/s41398-020-01008-9 PMC7527452

[41] Zhu J Peng Q Xu Y. Morinda officinalis oligosaccharides ameliorate depressive-like behaviors in poststroke rats through upregulating GLUT3 to improve synaptic activity. FASEB J. 2020;34:13376–95. 32812265 10.1096/fj.201902546RR

[42] Chen X Zhang G. Progress and prospects of acupuncture research on the mechanism of post-stroke depression. World J Acupunct Moxibustion. 2020;30:285–7.

[43] Zhong S Jianbin O Hua L. Clinical application and mechanism of acupuncture in the treatment of post-stroke depression. Inner Mongolia J Tradit Chin Med. 2022;41:142–4.

[44] Yang NN Lin LL Li YJ. Potential mechanisms and clinical effectiveness of acupuncture in depression. Curr Neuropharmacol. 2022;20:738–50. 35168522 10.2174/1570159X19666210609162809 PMC9878952

[45] Xiaoling L Jingxian W Ang L. Advances in brain fMRI research of scalp acupuncture therapy. Chin J Magn Reson Imaging. 2021;12:98–100 + 112.

[46] Siyu X Zhongren S Guangyue X. Progress on the mechanism of neurological function reconstruction after stroke treated with head acupuncture. J Emerg Tradit Chin Med. 2023;32:937–40.

[47] Zhang R Lao L Ren K. Mechanisms of acupuncture-electroacupuncture on persistent pain. Anesthesiology. 2014;120:482–503. 24322588 10.1097/ALN.0000000000000101 PMC3947586

